# Pattern and nature of Neyshabur train explosion blast injuries

**DOI:** 10.1186/s13017-018-0164-7

**Published:** 2018-01-19

**Authors:** Katayoun Jahangiri, Hasan Ghodsi, Ali Khodadadizadeh, Sadegh Yousef Nezhad

**Affiliations:** Department of Health in Disasters and Emergencies, School of Health, Safety and Environment, Shahid Beheshti University of Medical Sciences, Tehran, Iran

**Keywords:** Accidents, Train explosion, Man-made crises

## Abstract

**Background:**

Explosions are classified as both man-made and complex accidents. Explosive events can cause serious damage to people, property, and the environment. This study aimed to investigate the pattern and nature of damage incurred to the victims of the Neyshabur Train Explosion.

**Methods:**

The current study is a descriptive cross-sectional study that was retrospectively performed on 99 individuals using census method and documents victims hospitalized due to the Neyshabur train disaster (February 2004) in 2016. In this study, different variables such as age, sex, type of injury, treatment, etc. were examined using a questionnaire and were analyzed using SPSS16.

**Results:**

The results showed that 50.5% of victims were males with mean age of 30.33 ± 4.27 years and most of them were in 20- to 40-year age group. A total of 98 victims were discharged after treatment, and 1 victim died due to the severity of injuries after 3 days of hospitalization. Second type of injuries caused by the explosion accounted for most of the injuries (55.6%), and most treatments (54.5%) were related to the specific field of orthopedics.

**Conclusion:**

Handling and transportation of fuels and chemicals via rail transport system is one of the potential hazards that threatens human life. The results showed that the highest numbers of victims were in 20- to 40-year age group, which is the age of economic efficiency. The prevention and reduction of human and financial losses resulting from accidents require proper national planning.

## Background

Explosions are classified as both man-made and complex accidents. Regardless of the cause of the explosion, explosive events can cause serious damage to a lot of people. The severity of injuries and damages caused by explosions depend on several factors, including the explosion occurred at the closed or roofed place, the amount of explosives, victims distance from the explosion, and the presence of other wastes at the site of the explosions [1].

The explosions cause multiple injuries classified in four groups (primary, secondary, tertiary and quaternary injuries). Each of these injuries may occur individually or in combination with other groups [2, 3].

The primary injuries occur as a result of rapid propagation of blast waves and affect air-filled body organs such as the lungs, ears, and hollow viscera of the digestive system (colon) [4]. Bowel perforation, hemorrhage, and mesenteric shear injuries are some consequences of primary blast injuries [5].The most common organ that is affected by blast injury is the ears [6]. If the eardrum is intact, there is little risk of damage to other air-filled body organs [7]. The lung is the second organ affected by primary injuries [8]. The most common cause of death following explosions is the second injuries. Objects that are thrown around cause secondary injuries that are often penetrating wounds. Head, neck, chest, abdomen, and extremities injuries constitute the most common types of second injuries [9]. The most common tertiary injuries are closed fractures and injuries of the brain. The uncommon injuries include joint dislocations and in some cases, amputation [2, 4, 10, 11]. Damage of buildings and streets can cause blunt trauma and crush injuries [8]. Quaternary injuries include all injuries that are not included in three injury classifications such as respiratory injuries, burns, breathing toxic gases such as carbon monoxide, and choking and crush of bodies [11]. This study aimed to investigate the pattern and nature of injuries incurred to the victims of the train explosion in Neyshabur.

### Short summary of the scenario

**Fig. 1:**
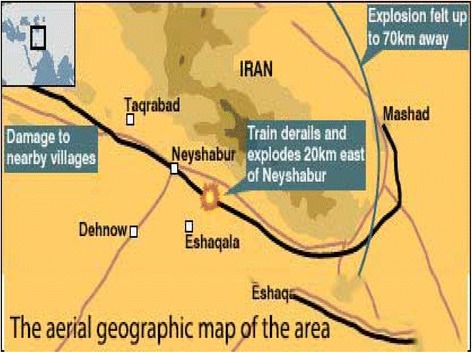
The aerial geographic map of the area

At 4:42 a.m. February 18, 2004, 51 wagons of a train that stopped at Abu Moslem Station started moving and then collided with a locomotive stopped at Khayyam Station, which led to the disarrangement of wagons and a primary fire (Fig. 1) [12]. The local firefighters from all the neighboring towns arrived to rescue anybody who might have been trapped inside and to extinguish several minor fires which had broken out in the wreckage [13].

At the beginning, the incident was a local event and Initial Command System the in scene was created by the chief of firefighters, then border railway homes were evacuated. The initial fire was controlled at 9 a.m., and the people returned to their home.

Local authorities and people went to watch the scene and thank the aid workers for their work. Unfortunately, at 9:37, suddenly, a very loud explosion occurred. Immediately after the explosion, revolutionary guards cordoned off a wide area around the disaster site overnight due to fears of further blasts and pollution [14].

### Description of hazard causing the accident

Fifty one wagons of a train carried sulfur, ammonium nitrate, cotton, and oil. In this incident, there were seven ammonium nitrate wagons with an approximate weight of 399 tons. After collision of wagons with a locomotive stopping at the wagon station, wagons collided and an initial fire occurred. Firefighters were not aware of wagons’ contents because fire diamond on the body of the wagons was not installed. An ammonium nitrate wagon was placed next to the flames and decomposed after 5 h and resulted in the explosion of other wagons [15].

### Total number and type of injuries

The explosion led to the death of more than 300 and injuries of more than 450 spectators, officials, and relief workers. A total of 24 of the firefighters, governor, firefighting director, and head of the city’s energy department died in this incident [12]. All people and animals, due to the severity of the explosion, died up to 500 m away [16]. The deceased victims were transferred to Neyshabur Forensic Medicine. The severity of the explosion was such that most of the bodies were disintegrated, and it was difficult to identify them. The injured people were transferred to Neyshabur and Mashhad hospitals by ambulance, personal, and military vehicles [12].

## Methods

The present study is a retrospective descriptive study that was conducted in 2016. The sample size was 99 subjects who with census method by reviewing the medical records of all the train explosion victims who were transferred to Neyshabur hospitals. After obtaining the necessary permits, researchers isolated medical records of train blast victims while visiting the medical records unit of hospitals. The instrument used in this study was two-part researcher-made questionnaire. The first part includes demographic information such as age, sex, marital status, place of residence, etc. The second part relates to the type of injury, type of treatment procedures, and the outcome of procedures. The data were entered into the software SPSS16 and then were described using frequency distribution tables and central and dispersion indicators.

## Results

The results of investigating all medical records in medical records unit of Neyshabur hospitals (Hakim and 22 Bahman hospitals) showed that although the number of injuries was reported by executive agencies to be over 450 people, there were only a total number of 99 medical records belonging to admitted victims in these medical centers. The average age of victims was 30.33 ± 4.27 years, and most of them (36.4%) were in the 40- to 20-year age group. The youngest and oldest victims were 1.5 and 76 years old, respectively. Some demographic characteristics of the research subjects are shown in Table 1.

**Table 1:** Some demographic characteristics of victims

Variable	Frequency	Percentage
Gender		
Male	50	50.5
Female	49	49.5
Marital status		
Married	61	61.6
Single	38	38.4
Hospital		
22 Bahman	72	72.7
Hakim	27	27.3

The majority of subjects (95%) were discharged from hospitals after the necessary treatment measures, and four patients (4%) were sent to the provincial capital after initial treatment measures for complementary therapies.

**Fig. 2:**
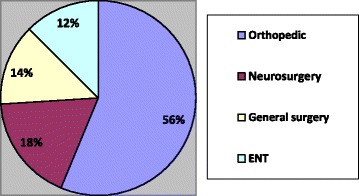
Frequency distribution of surgical procedures

The reason stated for sending these patients was lung trauma and the absence of thoracic specialist in Neyshabur hospitals in 2003. Despite treatment procedures, one of the victims died after 3 days of hospitalization due to severe injuries (hem thorax, pneumothorax, and severe pulmonary contusion). Most treatments were performed by the departments of orthopedics (56%), neurosurgery (18%), general surgery (14%), and ENT (12%) (Fig. 2). Minimum and maximum days of hospitalization were 1 and 57 days, respectively (average 4.27 ± 6.07 days).

The second type injuries accounted for more injuries imposed on hospitalized victims (55.6%). Information on the types of injuries and the number of victims is shown in Table 2.

**Table 2:** Frequency of type of injuries incurred on Neyshabur train disaster victims

Type of injury	Frequency	Percent
Primary injuries	11	11.1
Secondary injuries	55	55.6
Tertiary injuries	29	29.3
Quaternary injuries	4	4
Total	99	100

## Discussion

The results showed that most of the victims of this accident were in 20- to 40-year age group, while most of the victims were children in Bashkir train blast [11]. The main reason for the age differences among victims is related to the type of accident in the two explosions. A freight train was exploded in Neyshabur train disaster with no passenger, while a freight train collided with a passenger train (mostly children) in the Bashkir train explosion. Many victims of Neyshabur train disaster belonged to 20- to 40-year age group because many of them who were present at the scene of the accident and had come to rescue the injured were young and middle-aged people. The results showed that the secondary injuries accounted for the most of the explosion-induced injuries imposed on hospitalized victims. Due to the severity of the blast, most people who were at 500 m away from the accident died. Also, most of those who survived and were transferred to hospitals were at a larger distance from the accident and were injured due to shrapnel hit their bodies. Furthermore, most of measures were taken by the Department of Orthopedic Surgery to remove shrapnel. This is despite the fact that most victims were hospitalized due to burns in Bashkir train explosion [11]. However, a ruptured eardrum, lungs injury, hem thorax, pneumothorax, contusion, and rupture of internal organs of the abdomen account for most of injuries in the most explosive events [17], due to the severity of the blast in Neyshabur train disaster, people with this type of injuries died at the scene of the accident; therefore, the second injuries accounted for the most of injuries. However, the results showed that there was no victim in the early hours of the accident; when officials have declared the fire was extinguished, the evacuated people returned their homes and some of them also decided to watch the scene, the explosion occurred, and many people were killed and injured. In a similar incident that occurred in Baltimore in 2013, the total number of the injured was four people and the main reason for low victim number of the accident was reported to be in-time evacuation of the surrounding homes and prohibiting people to enter the scene of the incident [18].

The pattern of injury that occurs with explosions is unique. In this study, secondary injuries were higher than other injuries. And quaternary injuries were minimum in rate.

In this incident, due to the enormous blast force, most of the victims died at once at the scene of the incident.

From the above, it can be concluded that the high rate of casualty in this incident was due to the following reasons: 1—not regarding the rules of carrying hazardous materials and no installation fire diamond on the body of wagons, as a result of the lack of awareness of the firefighters and their mistake, and the decision to shut off the fire with water; 2—not protecting the perimeter of hot zone by the police and entry of unnecessary people to the scene of the incident.

## Conclusion

Since the consequences of man-made and natural disasters are not predictable; therefore, to better manage events and prevent crises in the future, ordinary people should be prevented from entering the scene, until the full assurance of the safety of the scene so that we can minimize the number of affected people in case of an explosion. The blast force causes very serious injuries and often leads to the death of people. Given the extent of the injuries incurred to those present at the scene of the explosion, diagnosis and treatment of injuries are difficult.

Recommendations for the future are as follows: 1—To prevent and better response to similar incident, local authorities must supply enough resources (such as experts, equipment, information, etc.). 2—To avoid mass casualties—if needed—Incident Commander should protect the environment of incident by the police and then send EMS, fire fighters, volunteers, and other responders to hot zone. 3—The appropriate communication between the organizations involved in the incident is needed. 4—Providing executive and operational instructions for its implementation during a crisis. 5—Emergency medical technicians should learn about response to explosive events such as intentional and non-intentional incidents. They should learn how to protect themselves from dangers in the scene. They should get familiar with the type of blast injuries for better performance. In this incident, unfortunately, complete data are not available in forensic medicine, EMS department, hospitals, and other organizations involved. So, it can be said that the documentation in this incident was very weak. So, for better lesson learned from the incident, documentation is very important.

### Limitation

Since this is a retrospective study, we could not get any other data such as reasons of mortality of people at the scene (cause of death and type of injuries of people who succumbed due to their injuries at scene that there were no on forensic medicine), trauma severity scores, detail about injuries frequency, and disability and the number of injured people who were treated by medical technicians on the scene.
